# Dynamic platelet profiles in the emergency department and their predictive value for 28-day all-cause mortality in septic patients

**DOI:** 10.1371/journal.pone.0348282

**Published:** 2026-05-06

**Authors:** Jianchun Wei, Xinjie Huang, Zhilian Wang, Hailing Zhang

**Affiliations:** 1 Department of Emergency Medicine, Aviation General Hospital, Beijing, China; 2 Department of General Surgery, Beijing Children’s Hospital, Capital Medical University, National Center for Children’s Health, Beijing, China; Tekirdag Namik Kemal University: Tekirdag Namik Kemal Universitesi, TÜRKIYE

## Abstract

**Background:**

Sepsis is a significant cause of global mortality and most frequently first presents in the emergency department. Dynamic platelet changes play a key pathophysiological role in sepsis, yet their comprehensive prognostic value—particularly from a multi-dimensional perspective that includes value, magnitude, and timing—remains underexplored.

**Methods:**

In this retrospective cohort study, 363 episodes of sepsis first diagnosed in the emergency department of Beijing Aviation General Hospital between April 2020 and January 2025 were included. We systematically analyzed three dimensions of platelet counts: 1) nadir value; 2) the magnitude of platelet decline; 3) dynamic timing (time to nadir and maximum decline). Multivariable Cox regression models were used to assess their independent associations with 28-day all-cause mortality, adjusting for demographics, comorbidities, infection characteristics, and disease severity.

**Results:**

Among 363 patients (median age 81 years, 54.5% male), the 28-day mortality rate was 39.9%. Compared to survivors, non-survivors had a lower platelet nadir [70.0 vs. 134 × 10⁹/L, *p* < 0.001] and a greater magnitude of platelet decline [62.8% vs. 31.5%, *p* < 0.001].Multivariable analysis revealed dose-response relationships for platelet nadir (moderate [50–99 × 10⁹/L]: aHR = 2.04, *p* = 0.014; severe [20–49 × 10⁹/L]: aHR = 2.98, *p* < 0.001; profound [<20 × 10⁹/L]: aHR = 3.46, *p* = 0.003) and magnitude of decline (moderate [30–50%]: aHR = 1.79, *p* = 0.044; severe [>50%]: aHR = 2.43, *p* < 0.001). Timing analysis demonstrated that intermediate and late platelet nadir were associated with higher mortality risk (intermediate: aHR = 4.39–7.28, all *p* < 0.001; late: aHR = 5.85–9.58, all *p* < 0.001), while initial and early nadir showed no significant association. Similarly, both intermediate and late maximum platelet decline were associated with increased mortality (intermediate: aHR = 2.70–2.98, all *p* < 0.001; late: aHR = 6.33–6.87, all *p* < 0.001).

**Conclusion:**

Through stepwise analysis, we demonstrate that dynamic platelet parameters—magnitude and timing of decline—independently predict 28-day mortality in sepsis. The magnitude applies to all patients but is static, while timing adds a critical dimension: the same degree of decline has different prognostic implications depending on when it occurs. Delayed decline and intermediate/late nadir indicate poor prognosis, whereas early nadir does not. These findings underscore the importance of longitudinal platelet monitoring in the emergency setting, as the trajectory—integrating both magnitude and timing—identifies at-risk patients, including those without thrombocytopenia.

## Introduction

Sepsis is a life-threatening organ dysfunction caused by a dysregulated host response to infection [1]. It remains a major global health issue with significant economic costs, with reported mortality rate as high as 26.7% [2–4].

Platelet (PLT) count is a routine and accessible laboratory parameter in sepsis management. Thrombocytopenia is observed in 35–59% of septic patients [5–7], resulting from mechanisms such as impaired production, increased consumption, microvascular sequestration, and immune-mediated destruction [8]. Beyond their role in coagulation, platelets are active immunomodulators: under physiological conditions, they help maintain endothelial integrity; in sepsis, they become activated, recognize pathogens via Toll-like receptors, and facilitate neutrophil extracellular trap (NET) formation to enhance pathogen clearance [9–13].

The role of PLT as a predictive factor for mortality remains controversial [14,15], especially early clinical data in the emergency department (ED) are not fully studied. We aim to analyze septic patients in the ED to investigate the association between dynamic platelet changes and 28-day all-cause mortality, specifically assessing: (1) whether platelet decline independently predicts mortality; (2) the dose-response relationship between the platelet nadir value and the magnitude of platelet decline with mortality risk; and (3) the prognostic value of the timing of nadir and maximum decline.

## Materials and methods

### Study design and participants

This retrospective cohort study consecutively included 363 episodes of sepsis first diagnosed in the Emergency Department of Aviation General Hospital between 03/04/2020 and 14/01/2025. Clinical data were retrospectively collected from electronic medical records between 12/08/2025 and 02/09/2025. Among these, 13 patients had two eligible visits, and 2 patients had three eligible visits during the study period; each visit was treated as an independent episode in the analysis.

**Inclusion criteria:**

1. Aged 18 years or older;
2. Meeting the Sepsis-3.0 diagnostic criteria, specifically the presence of a documented or suspected infection with an associated increase in the Sequential Organ Failure Assessment (SOFA) score of 2 points or greater [1,16,17].

**Exclusion criteria were as follows:**

1. Transplant recipients, given that long-term immunosuppressive therapy can induce bone marrow suppression and thrombocytopenia, thereby potentially confounding the primary exposure;
2. Pre-existing immune thrombocytopenia (ITP), thrombotic thrombocytopenic purpura (TTP), or other congenital/acquired thrombocytopenia disorders unrelated to sepsis;
3. Hematologic malignancies, or active solid tumors undergoing chemotherapy (due to potential chemotherapy-induced thrombocytopenia); patients with solid tumors on targeted therapy, those who were post-surgery and stable, or those not yet receiving treatment were not excluded;
4. COVID-19 diagnosed by a positive PCR test at the time of ED presentation. During the COVID-19 pandemic period (2020–2022), all patients presenting with sepsis symptoms were routinely tested for SARS-CoV-2 upon ED admission; after this period, testing was no longer routine. Patients were excluded based on active infection at presentation, not on prior history of COVID-19. Patients with COVID-19 were excluded because SARS-CoV-2 infection has been shown to be independently associated with thrombocytopenia [18,19], which could confound the primary exposure of sepsis-associated thrombocytopenia. Additionally, COVID-19 cases in our center were concentrated during a specific pandemic period (November 2022 to February 2023), and including these patients might introduce temporal bias;
5. Death within 24 hours of ED admission, as these patients had insufficient time for platelet dynamics to be meaningfully assessed;
6. Incomplete medical records, defined as any of the following:
  - Patients transferred from other hospitals whose baseline platelet count at the time of ED presentation could not reliably reflect their pre-admission status due to prior treatment that may have affected platelet levels; patients transferred from other hospitals without such treatment were included.
  - Patients with insufficient platelet measurements (e.g., only one or two samples during hospitalization), which precluded assessment of platelet dynamics.
  - Missing data for SOFA score calculation within 24 hours of ED admission, precluding severity assessment.

### Ethical approval

This retrospective medical record study involving human participants was reviewed and approved by the Ethics Committee of Aviation General Hospital (approval No. HK2025−57) on 11/08/2025. The requirement for informed consent was waived by the ethics committee because the data were analyzed anonymously and retrospectively.

### Data collection and variables

Demographic, clinical, and laboratory data were systematically collected from electronic medical records for all patients.

#### Demographic and baseline characteristics.

Age, gender, and comorbidities required for Charlson Comorbidity Index (CCI) calculation [20] were recorded. For analysis, CCI was evaluated both as a continuous variable (per 1-point increase) and as a categorical variable (0–1, 2–3, and ≥4 points) to capture potential nonlinear effects of comorbidity burden [21,22].

In addition, liver cirrhosis [23,24] and end-stage renal disease (ESRD) [25,26] were explicitly recorded as individual comorbidities due to their potential impact on platelet counts and clinical outcomes.

#### Clinical and laboratory parameters.

Vital signs (temperature, heart rate, respiratory rate, mean arterial pressure), Glasgow Coma Scale (GCS) score, oxygen saturation (SpO₂), fraction of inspired oxygen (FiO₂) were collected.

Laboratory measurements comprised platelet count, white blood cell (WBC) count, hemoglobin (Hb), hematocrit (Hct), C-reactive protein (CRP), procalcitonin (PCT), lactate (Lac), prothrombin time (PT), fibrinogen (FIB), D-dimer, arterial blood gas parameters (pH, partial pressure of oxygen [PaO₂]), serum creatinine (Cr), total bilirubin (TBil), blood glucose (Glu), sodium (Na⁺), and potassium (K⁺).

#### Clinical scores.

Several clinical scoring systems were assessed upon initial ED presentation. Organ dysfunction was quantified using the SOFA score [27], and overall disease severity was assessed using the APACHE II score [28]. Coagulopathy was evaluated using the ISTH-DIC score [29].

#### Site of infection and pathogens.

Infection sites were classified as respiratory tract, urinary tract, abdominal, or other sites (including skin and soft tissue, central nervous system, and bloodstream infections). Pathogens were categorized as Gram-positive bacteria, Gram-negative bacteria, or other pathogens (including fungi, viruses, and atypical pathogens).

#### High-risk antibiotic identification and quantification.

This study collected information on all antibiotics administered to each patient. To control for the confounding effect of antibiotics on thrombocytopenia, we classified antibiotics into high-risk and low-risk categories based on the diagnostic criteria for drug-induced thrombocytopenia established by George et al [30].

High-risk antibiotics were defined as those meeting either of the following criteria:

- Level I evidence: Case reports demonstrating that platelet counts normalized after drug discontinuation and decreased again upon re-challenge.
- Level II evidence: Multiple case series showing platelet recovery after drug withdrawal with no alternative explanation.

Based on these criteria, the following antibiotics were classified as high-risk: Vancomycin [31,32]; Piperacillin-tazobactam [33,34]; Meropenem [35]; Imipenem [36]; Ceftazidime [37]; Ceftriaxone [38]; Linezolid [39].

Low-risk antibiotics were defined as all other antibiotics not meeting the Level I/II criteria. It is important to note that “low-risk” does not imply “zero risk”; rather, it indicates that based on current evidence, there is insufficient support to classify them as high-risk according to the George criteria.

For each patient, the number of high-risk antibiotics used during hospitalization was calculated and included as a covariate in the multivariate analysis.

#### Time windows for laboratory measurements.

To ensure precision in our analysis, we defined measurement time points based on hours from ED admission, rather than calendar days. Clinical and laboratory parameters were serially assessed at five predefined time windows:

1. Baseline window: 0–24 hours after ED admission (Day 0);
2. Early window: 24–72 hours after ED admission (Days 1–3);
3. Intermediate window I: 72–120 hours after ED admission (Days 4–5);
4. Intermediate window II: 120–168 hours after ED admission (Days 6–7);
5. Late window: ≥ 168 hours after ED admission (Day ≥ 8).

If a patient had multiple measurements within a given time window, the worst value within that window was used for analysis (e.g., the lowest platelet count for platelet-related analyses, the highest white blood cell count for infection assessment). This definition was applied to all laboratory parameters.

### Definitions of platelet parameters

#### Nadir platelet count and magnitude of decline.

The nadir platelet count was defined as the lowest platelet value recorded during hospitalization.

The magnitude of platelet decline was calculated using the following formula:


Magnitude of platelet decline (%)=[(baseline platelet count−nadir platelet count)/baseline platelet count]×100%.

where baseline platelet count was defined as the first measurement obtained within 24 hours of ED admission (Day 0).

#### Platelet nadir groups.

Thrombocytopenia was defined as a platelet count ≤150 × 10⁹/L [40,41]. To assess its severity–outcome relationship in sepsis, patients were stratified, aligning with the platelet criteria of the SOFA score, into five grades based on the nadir platelet count:

1. normal group (PLT ≥ 150 × 10⁹/L);
2. mild reduction group (100 × 10⁹/L ≤ PLT < 150 × 10⁹/L);
3. moderate reduction group (50 × 10⁹/L ≤ PLT < 100 × 10⁹/L);
4. severe reduction group (20 × 10⁹/L ≤ PLT < 50 × 10⁹/L);
5. profound reduction group (PLT < 20 × 10⁹/L).

#### Magnitude of platelet decline groups.

Building upon the calculated maximum platelet decline, this study adapted the stratification rationale of the heparin-induced thrombocytopenia “4Ts” pretest score and incorporated an adjustment for the floor-effect [42]. Patients were subsequently classified into four distinct groups based on the magnitude of platelet decline, with clear clinical relevance:

1. stable group (decline ≤30% and baseline platelet count ≥50 × 10⁹/L);
2. floor-effect group (decline ≤30% and baseline platelet count <50 × 10⁹/L);
3. moderate decline group (30% < decline ≤50%);
4. severe decline group (decline >50%).

This classification refines the conventional “non-significant decline” category (≤30%) by distinguishing true physiological stability from decline masked by low baseline levels. Excluding patients with baseline thrombocytopenia from the stable group ensures a pure reference category and reduces floor-effect bias. The 50 × 10⁹/L baseline cutoff was adopted based on previous literature and preliminary data from this study [40,41].

#### Phases of platelet dynamics analysis.

Based on the predefined sampling time windows, the observation period was stratified into three sequential phases for the analysis of platelet dynamics:

1. Early phase (Days 0–3, combining baseline and early windows);
2. Intermediate phase (Days 4–7, combining intermediate windows I and II);
3. Late phase (Day ≥ 8, corresponding to the late window).

This division allowed phase-specific analysis of platelet dynamics and their prognostic relevance [40,43,44].

#### Timing of nadir platelet groups.

To evaluate the prognostic significance of platelet nadir timing, patients were categorized into five groups based on the timing of their platelet nadir:

1. normal group: patients with platelet count consistently≥150 × 10⁹/L;
2. initial nadir group: patients whose nadir occurred on Day 0;
3. early nadir group: patients whose nadir occurred on Day 1;
4. intermediate nadir group: patients whose nadir occurred on Day 3 or Day 5;
5. late nadir group: patients whose nadir occurred on Day 7.

These categories were designed for time-dependent Cox regression analysis. By defining nadir timing based on specific days of occurrence, the analysis inherently conditions on survival to that time point—patients must have survived long enough for their nadir to be observed at Day 1, Day 3/5, or Day 7. This approach ensures that only patients who survived to the day of nadir contribute to the post-nadir observation period, thereby avoiding immortal time bias.

#### Timing of maximum platelet decline groups.

To capture both the extent and timing of platelet deterioration, patients were categorized into five groups based on the magnitude and timing of their maximum platelet decline:

1. stable group: decline ≤30% and baseline platelet count ≥50 × 10⁹/L;
2. floor-effect group: decline ≤30% and baseline platelet count <50 × 10⁹/L;
3. early decline group: decline >30% with maximum decline occurring ≤3 days;
4. intermediate decline group: decline >30% with maximum decline occurring 4–7 days;
5. late decline group: decline >30% with maximum decline occurring ≥8 days.

These categories were designed for time-dependent Cox regression analysis. using a start–stop structure. Patients were considered unexposed (reference: stable group) before the occurrence of maximum platelet decline and transitioned to their corresponding group thereafter. By defining exposure status based on the timing of maximum decline, this approach ensures that only patients who survived to the day of maximum decline contribute to the exposed observation period, thereby avoiding immortal time bias.

### Severity adjustment and time-updated organ dysfunction assessment

To account for disease severity in our multivariable models, we selected the SOFA score as the primary measure of organ dysfunction. Although the APACHE II score is a well-validated prognostic tool in sepsis and showed a strong univariable association with mortality in our cohort, we prioritized SOFA for several reasons. First, SOFA is a core component of the Sepsis-3 definition and is specifically designed to quantify organ dysfunction, which aligns closely with our focus on platelet dynamics as a reflection of organ-specific pathophysiology. Second, given our objective to evaluate simple, accessible parameters for early risk stratification in the emergency setting, we aimed to maintain parsimony in model construction while capturing the most clinically relevant dimension of severity—organ dysfunction.

Baseline organ dysfunction was assessed using the full SOFA score calculated on Day 0 (0–24 hours after ED admission). This baseline SOFA score was included as a fixed covariate in all multivariable Cox regression models (Tables 3–6) to adjust for initial disease severity.

To account for time-varying organ dysfunction, we constructed a time-updated partial SOFA score (platelet count, total bilirubin, serum creatinine) for three phases: early (Days 1–3), intermediate (Days 4–7), and late (Day ≥ 8). For analyses of platelet nadir timing (Table 5) and maximum decline timing (Table 6), patients were assigned the partial SOFA score corresponding to the phase of their platelet event. This ensures concurrent assessment of organ dysfunction, while baseline severity was captured by the full SOFA score within 24 hours of ED admission (Day 0).

### Statistical analysis

Continuous variables were tested for normality using the Kolmogorov–Smirnov test. Normally distributed data are presented as mean ± standard deviation (SD) and compared using the independent samples t-test. Non-normally distributed data are expressed as median with interquartile range (IQR) and compared using the Mann–Whitney U test. Categorical variables are summarized as frequency (percentage) [n (%)] and compared using the chi-square (χ²) test or Fisher’s exact test when more than 20% of expected cell counts were <5.

#### Univariate Cox regression analysis.

Univariate Cox proportional hazards regression analysis was performed to evaluate the association between each candidate variable and 28-day mortality. Results are presented as hazard ratio (HR) with 95% confidence interval (CI). Variables with a univariate *p*-value < 0.1 or those considered clinically relevant were selected as candidates for multivariable analysis.

#### Kaplan–Meier survival curves.

**Fig 1:**
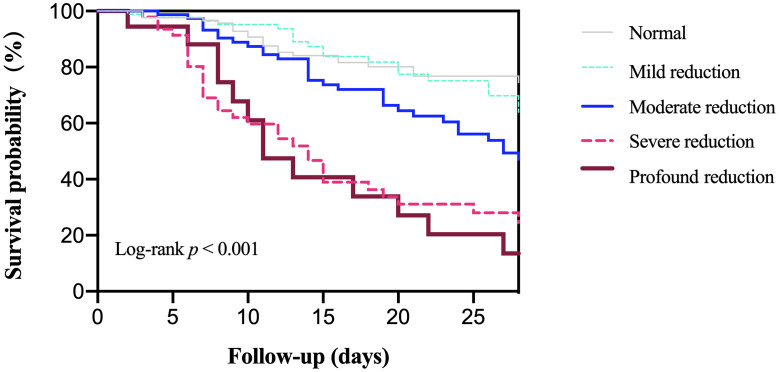
Association between platelet nadir group and 28-day all-cause mortality.

**Fig 2:**
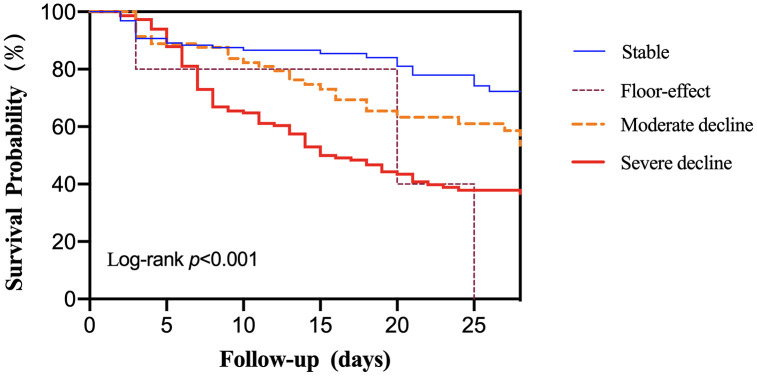
Association between magnitude of platelet decline and 28-day all-cause mortality.

**Fig 3:**
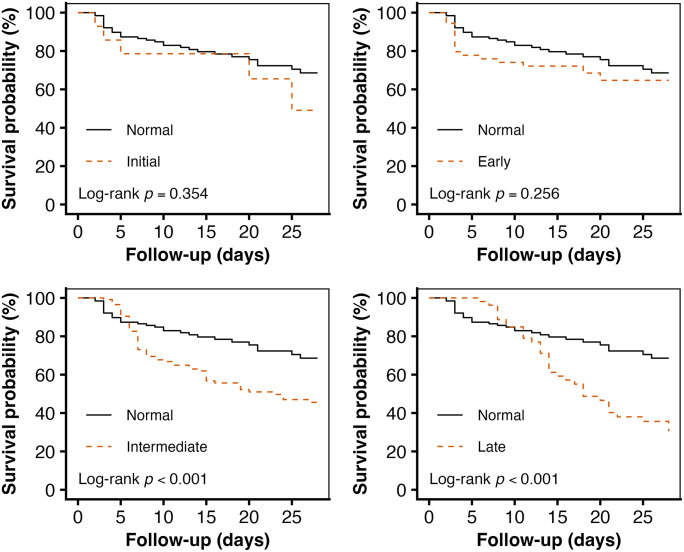
Association between timing of nadir platelet group and 28-day all-cause mortality.

**Fig 4:**
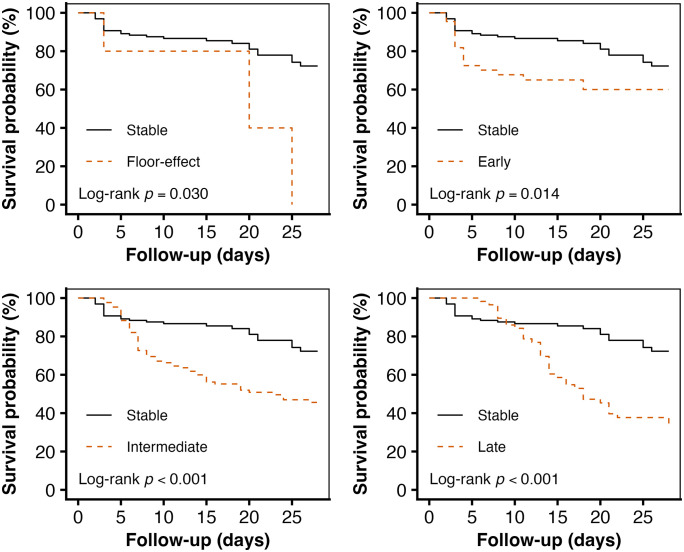
Association between timing of maximum platelet decline group and 28-day all-cause mortality.

Survival curves for different platelet parameter groups were estimated using the Kaplan–Meier method and compared using the log-rank test. These analyses were performed to visually illustrate the unadjusted association between platelet dynamics and 28-day mortality (Figs 1–4).

#### Multivariable Cox regression analysis for platelet nadir and magnitude of decline.

To evaluate the independent association of platelet nadir categories and magnitude of platelet decline with 28-day mortality, we constructed separate multivariable Cox proportional hazards models for each exposure (Tables 3 and 4). Covariates were selected based on univariable screening (*p* < 0.1) or clinical relevance, including demographics (age, sex), comorbidities (CCI), infection characteristics (pathogen type, number of high-risk antibiotics used), and disease severity markers (baseline SOFA score, lactate level, ISTH-DIC score, and baseline platelet count). To assess the robustness of the associations, we built three hierarchical models with progressive adjustment:

Model 1: adjusted for age and CCI;
Model 2: further adjusted for infection-related factors (pathogen type and number of high-risk antibiotics used);
Model 3: additionally adjusted for baseline disease severity markers (SOFA score, lactate level, ISTH-DIC score) and baseline platelet count.

Variance inflation factor (VIF) diagnostics were performed to assess multicollinearity among covariates. The proportional hazards assumption was verified using Schoenfeld residuals.

#### Time-dependent analysis for platelet nadir timing.

To evaluate the prognostic significance of platelet nadir timing, time-dependent Cox proportional hazards models were constructed using the five predefined nadir groups (normal, initial, early, intermediate, and late) as described in the Definitions section. The nadir timing was treated as a time-dependent categorical exposure variable to avoid immortal time bias.

Five hierarchical models were constructed:

Model 1: adjusted for age and CCI;
Model 2: further adjusted for infection-related factors, including Gram-positive infection, Gram-negative infection, other pathogens (including fungi, viruses, and atypical pathogens), and number of high-risk antibiotics used;
Model 3: additionally adjusted for baseline platelet count, baseline SOFA score, baseline lactate level, and baseline ISTH-DIC score;
Model 4: adjusted for covariates in Model 2 plus time-updated partial SOFA score and baseline platelet count, lactate, and ISTH-DIC score;
Model 5: adjusted for covariates in Model 2 plus time-updated partial SOFA score and baseline platelet count.

#### Time-dependent analysis for timing of maximum platelet decline.

To evaluate the prognostic value of the timing of maximum platelet decline, time-dependent Cox proportional hazards models were constructed using a start–stop structure based on the five predefined maximum decline groups (stable, floor-effect, early, intermediate, and late) as described in the Definitions section. Patients were considered unexposed (reference: stable group) before the occurrence of maximum platelet decline and transitioned to their corresponding group thereafter. Five hierarchical models were constructed following the same adjustment strategy as described for platelet nadir timing analysis (Models 1–5).

Results of multivariable analyses are presented as adjusted hazard ratios (aHR) with 95% CI. All tests were two-sided, and a *p*-value < 0.05 was considered statistically significant.

Descriptive statistics (Table 1), univariate analyses (Table 2), and multivariable Cox regression models with fixed covariates (Tables 3, 4) were performed using SPSS software (version 26.0; IBM Corp., Armonk, NY, USA). Time-dependent Cox regression analyses for platelet nadir timing and maximum platelet decline timing (Tables 5, 6) were performed using R software (version 4.5.1; R Foundation for Statistical Computing, Vienna, Austria).

## Results

This study included 363 septic patients with a median age of 81 (IQR 71–87) years; 198 (54.5%) were male. Based on 28-day outcomes, 145 patients (39.9%) were non-survivors.

Baseline characteristics were generally comparable between survivors and non-survivors, including age, sex, CCI, and the prevalence of liver cirrhosis and ESRD (all *p* > 0.05, Table 1). As expected, non-survivors exhibited greater disease severity, with significantly higher SOFA scores, APACHE II scores, lactate levels, and ISTH-DIC scores (all *p* < 0.001). Infection profiles and antibiotic exposure differed between groups.

**Table 1:** Baseline characteristics and clinical outcomes of septic patients stratified by 28-day all-cause mortality status.

Characteristic	Total (n = 363)	Survivors (n = 218)	Non-survivors (n = 145)	Statistical Value	*p*-value
**Demographics**					
Age, years	81 (71, 87)	80 (70, 87)	82 (72, 87)	−1.583	0.113
Male sex, n (%)	198 (54.5)	119 (54.6)	79 (54.5)	<0.001	0.984
**Comorbidities**					
CCI score, points	2 (1,4)	2 (1,4)	3 (1,5)	−1.695	0.090
CCI 0–1 points, n (%)	115 (31.7)	75 (34.4)	40 (27.6)	2.063	0.356
CCI 2–3 points, n (%)	115 (31.7)	68 (31.2)	47 (32.4)		
CCI ≥ 4 points, n (%)	133 (36.6)	75 (34.4)	58 (40.0)		
Liver cirrhosis ^a^, n (%)	3 (0.8)	1 (0.5)	2 (1.4)	–	0.566
ESRD, n (%)	23 (6.3)	11 (5.0)	12 (8.3)	1.531	0.216
**Site of Infection, n (%)**				8.389	0.039
Respiratory tract	214 (58.9)	120 (55.1)	94 (64.8)		
Urinary tract	53 (14.6)	39 (17.9)	14 (9.7)		
Abdominal	76 (20.9)	50 (22.9)	26 (17.9)		
Other infection sites ^b^	20 (5.5)	9 (4.1)	11 (7.6)		
**Pathogens, n (%)**					
Any pathogen detected	276 (76.0)	166 (76.1)	110 (75.9)	0.004	0.950
Gram-positive	89 (24.5)	61 (28.0)	28 (19.3)	3.538	0.060
Gram-negative	214 (58.9)	138 (63.3)	76 (52.4)	4.267	0.039
Other pathogens ^c^	65 (17.9)	31 (14.2)	34 (23.4)	5.045	0.025
**Antibiotic Exposure**					
High-risk antibiotic use, n (%)	251 (69.1)	144 (66.1)	107 (73.8)	2.444	0.118
Number of high-risk antibiotics used	1 (0, 2)	1 (0, 1)	1 (0, 1)	−1.430	0.153
**Disease Severity Parameters**					
SOFA score, points	6 (5, 9)	6(4,8)	8(6,10)	−5.649	<0.001
APACHE II score, points	21 (15, 26)	19 (14, 24)	25 (17, 29)	−5.015	<0.001
Lactate, mmol/L	2.3 (1.2, 4.0)	1.8 (1.0, 3.2)	2.8 (1.5, 5.6)	−4.295	<0.001
ISTH-DIC score, points	0(0, 3)	0 (0, 2)	1 (0, 3)	−3.539	<0.001
**Platelet Parameters**					
Nadir platelet count, × 10⁹/L	116(59, 180)	134 (93, 200)	70 (36, 134)	−7.016	<0.001
Magnitude of platelet decline, %	40.5(20.3, 64.7)	31.5 (16.0, 52.4)	62.8 (33.8, 80.8)	−7.030	<0.001

1.Data are presented as median (interquartile range) for continuous variables and n (%) for categorical variables.

2.Group comparisons were performed using the Mann‑Whitney U test for continuous variables and the χ² test or Fisher’s exact test for categorical variables, as appropriate.

3.^a^ Fisher’s exact test due to small sample size (n = 3).

4.^b^ Other infection sites included skin and soft tissue, central nervous system, and bloodstream infections.

5.^c^ Other pathogens included fungi, viruses, and atypical pathogens.

6.High-risk antibiotics were defined based on George criteria (Level I/II evidence) and included vancomycin, piperacillin-tazobactam, meropenem, imipenem, ceftazidime, ceftriaxone, and linezolid. The number of high-risk antibiotics used during hospitalization was calculated for each patient.

Abbreviations: CCI, Charlson Comorbidity Index; ESRD, end-stage renal disease; SOFA, Sequential Organ Failure Assessment; APACHE II, Acute Physiology and Chronic Health Evaluation II; ISTH-DIC, International Society on Thrombosis and Haemostasis Disseminated Intravascular Coagulation.

A core finding of this study was the pronounced disparity in platelet profiles. Non-survivors exhibited a significantly lower platelet nadir [70.0 (IQR 36–134) vs 134 (IQR 93–200) × 10⁹/L; *p* < 0.001] and a greater magnitude of platelet decline [62.8% (IQR 33.8–80.8) vs 31.5% (IQR 16.0–52.4); *p* < 0.001] compared to survivors (Table 1).

To assess the association between platelet parameters (nadir and Magnitude of decline) and 28-day mortality, we performed univariable Cox proportional hazards regression. Survival time was calculated from admission to death or censoring. Results are presented as HRs with 95% CIs in Table 2.

**Table 2:** Crude Univariate Cox regression analysis of factors associated with 28-day all-cause mortality in sepsis patients.

Characteristic	HR	95% CI	*p*-value
**Demographics**			
Age, per 5-year increase	1.02	0.96–1.09	0.545
Male sex	0.98	0.71–1.36	0.917
**Comorbidities**			
CCI score (continuous), per 1 point	1.03	0.96–1.11	0.403
CCI score (categorized)			
CCI 0–1 point	Reference	–	–
CCI 2–3 points	1.10	0.72–1.68	0.648
CCI ≥ 4 points	1.15	0.77–1.71	0.510
Liver cirrhosis ^a^	1.60	0.40–6.46	0.510
ESRD	1.42	0.79–2.57	0.241
**Site of Infection**			
Respiratory tract	1.22	0.87-1.71	0.256
Urinary tract	0.61	0.35-1.07	0.083
Abdominal	0.94	0.62-1.44	0.783
Other infection sites ^b^	1.25	0.68-2.32	0.470
**Pathogens**			
Any pathogen detected	0.74	0.51-1.09	0.125
Gram-positive	0.61	0.40-0.92	0.019
Gram-negative	0.54	0.39-0.75	<0.001
Other pathogens ^c^	1.45	0.90-2.13	0.057
**Antibiotic Exposure**			
High-risk antibiotic use	1.21	0.84–1.75	0.310
Number of high-risk antibiotics used, per 1 antibiotic	1.06	0.87–1.30	0.552
**Disease Severity Parameters**			
SOFA score, per 1 point	1.14	1.09-1.20	<0.001
APACHE II score, per 1 point	1.06	1.03–1.08	<0.001
Lactate, per 1 mmol/L	1.10	1.06–1.15	<0.001
ISTH-DIC score, per 1 point	1.20	1.08-1.32	<0.001
**Platelet Parameters**			
Baseline platelet count, per 10 × 10⁹/L increase	1.00	0.98–1.01	0.581
Nadir platelet count, per 10 × 10⁹/L increase	0.94	0.92–0.96	<0.001
Magnitude of platelet decline, per 10% increase	1.23	1.16–1.32	<0.001

1.Data are presented as hazard ratio (HR) and 95% confidence interval (CI). All variables were analyzed by univariable Cox proportional hazards regression.

2.HRs presented are crude (unadjusted) estimates.

3.Continuous variables were modeled as linear terms.

4.^a^ Only 3 patients (0.8% of the cohort) had liver cirrhosis; the estimate for this variable is unstable and should be interpreted with caution.

5.^b^ Other infection sites included skin and soft tissue, central nervous system, and bloodstream infections.

6.^c^ Other pathogens included fungi, viruses, and atypical pathogens.

7.High-risk antibiotics were defined based on George criteria (Level I/II evidence) and included vancomycin, piperacillin-tazobactam, meropenem, imipenem, ceftazidime, ceftriaxone, and linezolid. The number of high-risk antibiotics used during hospitalization was calculated for each patient.

Abbreviations: CCI, Charlson Comorbidity Index; ESRD, end-stage renal disease; SOFA, Sequential Organ Failure Assessment; APACHE II, Acute Physiology and Chronic Health Evaluation II; ISTH-DIC, International Society on Thrombosis and Haemostasis Disseminated Intravascular Coagulation.

Among the platelet parameters, nadir platelet count (HR 0.94, *p* < 0.001) and magnitude of platelet decline (HR 1.23, *p* < 0.001) were significantly associated with 28-day mortality. Baseline platelet count showed no significant association (*p* = 0.581).

Regarding pathogen type, Gram-positive (HR 0.61, *p* = 0.019) and Gram-negative (HR 0.54, *p* < 0.001) detection were unexpectedly associated with lower mortality, while other pathogens showed a non-significant trend toward increased risk (HR 1.45, *p* = 0.057).

For antibiotic exposure, neither high-risk antibiotic use (HR 1.21, 95% CI 0.84–1.75, *p* = 0.310) nor the number of high-risk antibiotics used (HR 1.06 per 1 antibiotic, 95% CI 0.87–1.30, *p* = 0.552) was significantly associated with 28-day mortality.

Based on platelet nadir levels, the 363 included patients were categorized into five groups: normal (n = 127), mild reduction (n = 87), moderate reduction (n = 84), severe reduction (n = 47), and profound reduction (n = 18). Kaplan-Meier survival curves demonstrated significantly divergent survival outcomes across these groups (log-rank *p* < 0.001; Fig 1).

To examine the independent association of platelet nadir groups with 28-day mortality, we constructed a series of multivariable Cox proportional hazards models with sequential adjustment for potential confounders (Table 3). Covariates were selected based on univariable screening (p < 0.1) and clinical relevance.

**Table 3:** Multivariable Cox regression analysis of the association between platelet nadir group and 28-day all-cause mortality.

Platelet Nadir Group	aHR (95% CI)	*p*-value
**Model 1: Adjusted for age and CCI**		
Normal (Reference)	1.00	**–**
Mild reduction	1.05 (0.61–1.79)	0.866
Moderate reduction	1.97 (1.23–3.15)	0.005
Severe reduction	3.48 (2.14–5.68)	<0.001
Profound reduction	3.92 (2.08–7.40)	<0.001
**Model 2: Model 1 + pathogen type and number of high-risk antibiotics**		
Normal (Reference)	1.00	–
Mild reduction	1.00 (0.59–1.72	0.994
Moderate reduction	1.98 (1.23–3.19)	0.005
Severe reduction	3.07 (1.86–5.06)	<0.001
Profound reduction	4.07 (2.09–7.91)	<0.001
**Model 3: Model 2 + SOFA, lactate, ISTH-DIC, and baseline platelet count**		
Normal (Reference)	1.00	–
Mild reduction	1.20 (0.67–2.17)	0.539
Moderate reduction	2.04 (1.16–3.59)	0.014
Severe reduction	2.98 (1.61–5.51)	<0.001
Profound reduction	3.46 (1.52–7.90)	0.003

1.Data are presented as adjusted hazard ratio (aHR) and 95% confidence interval (CI). All variables were analyzed by multivariable Cox proportional hazards regression.

2.Continuous variables were modeled as linear terms. Age was analyzed as per 5-year increase; baseline platelet count was analyzed as per 10 × 10⁹/L increase; number of high-risk antibiotics used during hospitalization was analyzed as per 1 antibiotic; CCI, SOFA score, lactate level, and ISTH-DIC score were analyzed as per 1-unit increase.

3.Categorical variables included pathogen type, with Gram-positive bacteria, Gram-negative bacteria, and other pathogens (including fungi, viruses, and atypical pathogens) entered as separate covariates in the model.

4.High-risk antibiotics were defined based on George criteria (Level I/II evidence) and included vancomycin, piperacillin-tazobactam, meropenem, imipenem, ceftazidime, ceftriaxone, and linezolid. The number of high-risk antibiotics used during hospitalization was calculated for each patient.

5.Platelet nadir groups were defined as: normal (≥150 × 10⁹/L), mild reduction (100–149 × 10⁹/L), moderate reduction (50–99 × 10⁹/L), severe reduction (20–49 × 10⁹/L), and profound reduction (<20 × 10⁹/L).

Abbreviations: CI, confidence interval; aHR, adjusted hazard ratio; CCI, Charlson Comorbidity Index; SOFA, Sequential Organ Failure Assessment; ISTH-DIC, International Society on Thrombosis and Haemostasis Disseminated Intravascular Coagulation.

Multicollinearity diagnostics confirmed no significant collinearity among covariates (all VIF < 5; range: 1.02–1.62), supporting their simultaneous inclusion in the multivariable model.

In the multivariable-adjusted model (Model 3), a pronounced dose-response relationship emerged between platelet nadir categories and 28-day mortality. Compared to the normal group, the mild reduction group did not show a statistically significant association with mortality risk (aHR = 1.20, 95% CI: 0.67–2.17, *p* = 0.539). However, the moderate reduction group exhibited a significantly elevated mortality risk (aHR = 2.04, 95% CI: 1.16–3.59, *p* = 0.014). This risk progressively increased with greater platelet reduction, reaching aHR = 2.98 (95% CI: 1.61–5.51, *p* < 0.001) in the severe reduction group and aHR = 3.46 (95% CI: 1.52–7.90, *p* = 0.003) in the profound reduction group.

To assess the dose-response relationship between the magnitude of platelet decline and mortality risk, patients were categorized into four predefined groups: stable (n = 129), floor-effect (n = 5), moderate decline (n = 81), and severe decline (n = 148). Kaplan-Meier analysis revealed significantly divergent survival outcomes across these groups (log-rank *p* < 0.001; Fig 2), demonstrating a clear dose-response relationship between the extent of platelet decline and mortality risk.

The independent association between magnitude of platelet decline and 28-day mortality was further evaluated using multivariable Cox regression with a sequential adjustment strategy as described for platelet nadir group (model 1–3, Table 4).

**Table 4:** Multivariable Cox regression analysis of the association between magnitude of platelet decline group and 28-day all-cause mortality.

Magnitude of Platelet Decline Group	aHR (95% CI)	*p*-value
**Model 1: Adjusted for age and CCI**		
Stable (Reference)	1.00	**–**
Floor-effect ^a^	3.66 (1.08–12.36)	0.037
Moderate decline	1.79 (1.05–3.04)	0.031
Severe decline	3.18 (2.05–4.95)	<0.001
**Model 2: Model 1 + pathogen type and number of high-risk antibiotics**		
Stable (Reference)	1.00	–
Floor-effect ^a^	2.98 (0.87–10.13)	0.081
Moderate decline	1.82 (1.07–3.11)	0.027
Severe decline	3.07 (1.95–4.83)	<0.001
**Model 3: Model 2 + SOFA, lactate, ISTH-DIC, and baseline platelet count**		
Stable (Reference)	1.00	–
Floor-effect ^a^	1.86 (0.48–7.17)	0.365
Moderate decline	1.79 (1.02–3.15)	0.044
Severe decline	2.43 (1.46–4.05)	0.001

1.Data are presented as adjusted hazard ratio (aHR) and 95% confidence interval (CI). All variables were analyzed by multivariable Cox proportional hazards regression.

2.Continuous variables were modeled as linear terms. Age was analyzed as per 5-year increase; baseline platelet count was analyzed as per 10 × 10⁹/L increase; number of high-risk antibiotics used during hospitalization was analyzed as per 1 antibiotic; CCI, SOFA score, lactate level, and ISTH-DIC score were analyzed as per 1-unit increase.

3.Categorical variables included pathogen type, with Gram-positive bacteria, Gram-negative bacteria, and other pathogens (including fungi, viruses, and atypical pathogens) entered as separate covariates in the model.

4.High-risk antibiotics were defined based on George criteria (Level I/II evidence) and included vancomycin, piperacillin-tazobactam, meropenem, imipenem, ceftazidime, ceftriaxone, and linezolid. The number of high-risk antibiotics used during hospitalization was calculated for each patient.

5.Magnitude of platelet decline was categorized as: stable group (decline ≤30% and baseline platelet count ≥50 × 10⁹/L), floor-effect group (decline ≤30% and baseline platelet count <50 × 10⁹/L), moderate decline group (30% < decline ≤50%), and severe decline group (decline >50%).

6.^a.^ The floor-effect group included only 5 patients (1.4% of the cohort); results should be interpreted with caution due to the small sample size.

Abbreviations: CI, confidence interval; aHR, adjusted hazard ratio; CCI, Charlson Comorbidity Index; SOFA, Sequential Organ Failure Assessment; ISTH-DIC, International Society on Thrombosis and Haemostasis Disseminated Intravascular Coagulation.

Multicollinearity diagnostics confirmed no significant collinearity among covariates (all VIF < 5; range: 1.02–1.33), supporting their simultaneous inclusion in the multivariable model.

In the multivariable-adjusted model (Model 3), a significant dose-response relationship was observed between platelet decline categories and 28-day mortality. Compared with the stable group (reference), the severe decline group showed a significantly increased risk of death (aHR = 2.43, 95% CI: 1.46–4.05, *p* < 0.001). The moderate decline group also exhibited a statistically significant association with higher mortality risk (aHR = 1.79, 95% CI: 1.02–3.15, *p* = 0.044). However, no significant association was observed for the floor-effect group (aHR = 1.86, 95% CI: 0.48–7.17, *p* = 0.365).

To further elucidate the dose-response relationship between platelet nadir timing and mortality, patients were categorized into five groups based on the timing of their platelet nadir: normal (n = 127), initial nadir (n = 14), early nadir (n = 54), intermediate nadir (n = 115), and late nadir (n = 53). A landmark analysis was performed to address potential immortal time bias, comparing 28-day mortality among the initial, early, intermediate, and late nadir groups with the normal group as the reference (Fig 3).

Multicollinearity diagnostics confirmed no significant collinearity among covariates prior to constructing the time-dependent Cox models (all VIF < 5; range: 1.02–1.27).

The independent association between platelet nadir timing and 28-day mortality was evaluated using multivariable time-dependent Cox regression with a sequential adjustment strategy (Table 5). In Model 1 (adjusted for age and CCI), both the intermediate nadir group (aHR = 4.39, 95% CI: 2.62–7.35, *p* < 0.001) and the late nadir group (aHR = 6.02, 95% CI: 3.40–10.65, *p* < 0.001) were associated with significantly increased mortality risk compared with the normal group. These associations remained consistent after further adjustment for infection-related factors in Model 2 (intermediate: aHR = 4.39, 95% CI: 2.61–7.39, *p* < 0.001; late: aHR = 6.09, 95% CI: 3.40–10.89, *p* < 0.001).

**Table 5:** Multivariable Cox regression analysis of the association between timing of nadir platelet group and 28-day all-cause mortality.

Timing of Nadir Platelet Group	aHR (95% CI)	*p*-value
**Model 1: Adjusted for age and CCI**		
Normal (Reference)	1.00	**–**
Initial nadir	2.12 (0.82–5.45)	0.119
Early nadir	1.81 (0.95–3.44)	0.071
Intermediate nadir	4.39 (2.62–7.35)	<0.001
Late nadir	6.02 (3.40–10.65)	<0.001
**Model 2: Model 1 + pathogen type and number of high-risk antibiotics**		
Normal (Reference)	1.00	–
Initial nadir	1.78 (0.69–4.60)	0.237
Early nadir	1.56 (0.81–2.98)	0.180
Intermediate nadir	4.39 (2.61–7.39)	<0.001
Late nadir	6.09 (3.40–10.89)	<0.001
**Model 3: Model 2 + SOFA, lactate, ISTH-DIC, and baseline platelet count**		
Normal (Reference)	1.00	–
Initial nadir	1.74 (0.61–4.97)	0.302
Early nadir	1.66 (0.81–3.41)	0.165
Intermediate nadir	4.50 (2.59–7.81)	<0.001
Late nadir	5.85 (3.20–10.70)	<0.001
**Model 4: Model 2 +** **time-updated partial SOFA score, lactate, ISTH-DIC, and baseline platelet count**		
Normal (Reference)	1.00	–
Initial nadir	1.31 (0.44–3.89)	0.623
Early nadir	1.32 (0.63–2.75)	0.456
Intermediate nadir	7.28 (3.99–13.30)	<0.001
Late nadir	9.57 (5.01–18.27)	<0.001
**Model 5: Model 2 +** **time-updated partial SOFA score + baseline platelet count**		
Normal (Reference)	1.00	–
Initial nadir	1.12 (0.38–3.25)	0.839
Early nadir	1.28 (0.62–2.65)	0.504
Intermediate nadir	7.16 (3.93–13.04)	<0.001
Late nadir	9.58 (5.02–18.26)	<0.001

1.Data are presented as adjusted hazard ratio (aHR) and 95% confidence interval (CI). All variables were analyzed by multivariable Cox proportional hazards regression with time-dependent covariates.

2.Continuous variables were modeled as linear terms. Age was analyzed as per 5-year increase; baseline platelet count was analyzed as per 10 × 10⁹/L increase; number of high-risk antibiotics used during hospitalization was analyzed as per 1 antibiotic; CCI, SOFA score, lactate level, ISTH-DIC score, and the time-updated partial SOFA score were analyzed as per 1-unit increase.

3.Categorical variables included pathogen type, with Gram-positive bacteria, Gram-negative bacteria, and other pathogens (including fungi, viruses, and atypical pathogens) entered as separate covariates in the model.

4.High-risk antibiotics were defined based on George criteria (Level I/II evidence) and included vancomycin, piperacillin-tazobactam, meropenem, imipenem, ceftazidime, ceftriaxone, and linezolid. The number of high-risk antibiotics used during hospitalization was calculated for each patient.

5.Patients were classified into five groups based on the timing of their platelet nadir:

normal group: patients with platelet count consistently≥150 × 10⁹/L (reference);

initial nadir group: patients whose nadir occurred on Day 0;

early nadir group: patients whose nadir occurred on Day 1;

intermediate nadir group: patients whose nadir occurred on Day 3 or Day 5;

late nadir group: patients whose nadir occurred on Day 7.

6.The time-updated partial SOFA score was constructed using three components of SOFA score: platelet count (coagulation), total bilirubin (liver), and serum creatinine (renal). This score was calculated at baseline (ED admission) and in the early, intermediate, and late phases of hospitalization, and was assigned to each patient based on the timing of their platelet nadir, thereby reflecting organ dysfunction occurring concurrently with the nadir.

Abbreviations: CI, confidence interval; aHR, adjusted hazard ratio; CCI, Charlson Comorbidity Index; SOFA, Sequential Organ Failure Assessment; ISTH-DIC, International Society on Thrombosis and Haemostasis Disseminated Intravascular Coagulation; ED, emergency department.

After additional adjustment for baseline disease severity markers and baseline platelet count in Model 3, the associations persisted (intermediate: aHR = 4.50, 95% CI: 2.59–7.81, *p* < 0.001; late: aHR = 5.85, 95% CI: 3.20–10.70, *p* < 0.001). In Model 4, which incorporated time-updated organ dysfunction scores, the associations became even stronger (intermediate: aHR = 7.28, 95% CI: 3.99–13.30, *p* < 0.001; late: aHR = 9.57, 95% CI: 5.01–18.27, *p* < 0.001). Similar results were observed in Model 5 with time-updated SOFA scores (intermediate: aHR = 7.16, 95% CI: 3.93–13.04, *p* < 0.001; late: aHR = 9.58, 95% CI: 5.02–18.26, *p* < 0.001).

To further elucidate the dose-response relationship between the timing of maximum platelet decline and 28-day mortality, patients were categorized into five groups based on the timing of platelet decline: (1) stable (n = 129); (2) floor-effect (n = 5); (3) early decline (n = 44); (4) intermediate decline (n = 128); (5) late decline (n = 57). A landmark analysis was performed to address potential immortal time bias, comparing 28-day mortality among the floor-effect, early, intermediate, and late decline groups with the stable group as the reference (Fig 4).

Multicollinearity diagnostics confirmed no significant collinearity among covariates prior to constructing the time-dependent Cox models (all VIF < 5; range: 1.02–1.31).

The independent association between timing of maximum platelet decline and 28-day mortality was evaluated using multivariable time-dependent Cox regression with sequential adjustment (Table 6). In Model 1(adjusted for age and CCI), both the intermediate decline (aHR = 2.98, 95% CI: 1.79–4.96, *p* < 0.001) and the late decline (aHR = 6.56, 95% CI: 3.61–11.89, *p* < 0.001) groups were associated with significantly increased mortality risk compared with the stable group. These associations remained consistent after further adjustment for infection-related factors in Model 2 (intermediate: aHR = 2.97, 95% CI: 1.77–4.97, *p* < 0.001; late: aHR = 6.87, 95% CI: 3.76–12.54, *p* < 0.001).

**Table 6:** Multivariable Cox regression analysis of the association between timing of maximum platelet decline group and 28-day all-cause mortality.

Timing of Maximum Platelet Decline Group	aHR (95% CI)	*p*-value
**Model 1: Adjusted for age and CCI**		
Stable (Reference)	1.00	**–**
Floor-effect ^a^	2.48 (0.58–10.54)	0.218
Early decline	1.27 (0.59–2.74)	0.545
Intermediate decline	2.98 (1.79–4.96)	<0.001
Late decline	6.56 (3.61–11.89)	<0.001
**Model 2: Model 1 + pathogen type and number of high-risk antibiotics**		
Stable (Reference)	1.00	–
Floor-effect ^a^	2.11 (0.49–9.00)	0.315
Early decline	1.13 (0.52–2.45)	0.765
Intermediate decline	2.97 (1.77–4.97)	<0.001
Late decline	6.87 (3.76–12.54)	<0.001
**Model 3: Model 2 + SOFA, lactate, ISTH-DIC, and baseline platelet count (per 10 × 10⁹/L increase)**		
Stable (Reference)	1.00	–
Floor-effect ^a^	0.80 (0.18–3.67)	0.778
Early decline	0.83 (0.38–1.82)	0.642
Intermediate decline	2.70 (1.60–4.59)	<0.001
Late decline	6.33 (3.42–11.70)	<0.001
**Model 4: Model 2 + time-updated partial SOFA score, lactate, ISTH-DIC, and baseline platelet count**		
Stable (Reference)	1.00	–
Floor-effect ^a^	0.87 (0.19–4.05)	0.863
Early decline	1.42 (0.63–3.21)	0.401
Intermediate decline	5.16 (2.88–9.26)	<0.001
Late decline	8.44 (4.44–16.04)	<0.001
**Model 5: Model 2 +** **time-updated partial SOFA score + baseline platelet count**		
Stable (Reference)	1.00	–
Floor-effect ^a^	1.08 (0.23–4.93)	0.924
Early decline	1.66 (0.74–3.71)	0.217
Intermediate decline	5.45 (3.04–9.79)	<0.001
Late decline	8.99 (4.73–17.06)	<0.001

1.Data are presented as adjusted hazard ratio (aHR) and 95% confidence interval (CI). All analyses were performed using multivariable Cox proportional hazards regression with time-dependent covariates.

2.Continuous variables were modeled as linear terms. age was analyzed as per 5-year increase; baseline platelet count was analyzed as per 10 × 10⁹/L increase; number of high-risk antibiotics used during hospitalization was analyzed as per 1 antibiotic; CCI, SOFA score, lactate level, ISTH-DIC score, and the time-updated partial SOFA score were analyzed as per 1-unit increase.

3.Categorical variables included pathogen type, with Gram-positive bacteria, Gram-negative bacteria, and other pathogens (including fungi, viruses, and atypical pathogens) entered as separate covariates in the model.

4.High-risk antibiotics were defined based on George criteria (Level I/II evidence) and included vancomycin, piperacillin-tazobactam, meropenem, imipenem, ceftazidime, ceftriaxone, and linezolid. The total number of such antibiotics used during hospitalization was calculated for each patient.

5.Patients were classified into five groups based on the magnitude and timing of their maximum platelet decline

stable group: decline ≤30% and baseline platelet count ≥50 × 10⁹/L (reference);

floor-effect group: decline ≤30% and baseline platelet count <50 × 10⁹/L;

early decline group: decline >30% and timing ≤3 days;

intermediate decline group: decline >30% and timing 4–7 days;

late decline group: decline >30% and timing ≥8 days.

a.The floor-effect group included only 5 patients (1.4% of the cohort); results should be interpreted with caution due to the small sample size.

6.The time-updated partial SOFA score was constructed using three components of the SOFA score: platelet count (coagulation), total bilirubin (liver), and serum creatinine (renal). This score was calculated at baseline (ED admission) and in the early, intermediate, and late phases of hospitalization, and was assigned to each patient based on the timing of their maximum platelet decline, thereby reflecting organ dysfunction occurring concurrently with the decline.

Abbreviations: CI, confidence interval; aHR, adjusted hazard ratio; CCI, Charlson Comorbidity Index; SOFA, Sequential Organ Failure Assessment; ISTH-DIC, International Society on Thrombosis and Haemostasis Disseminated Intravascular Coagulation; ED, emergency department.

After additional adjustment for baseline disease severity markers and platelet count in Model 3, the associations remained highly significant (intermediate: aHR = 2.70, 95% CI: 1.60–4.59, *p* < 0.001; late: aHR = 6.33, 95% CI: 3.42–11.70, *p* < 0.001). In Model 4, which incorporated time-updated organ dysfunction scores, the associations became even stronger (intermediate: aHR = 5.16, 95% CI: 2.88–9.26, *p* < 0.001; late: aHR = 8.44, 95% CI: 4.44–16.04, *p* < 0.001). Similar results were observed in Model 5 with time-updated SOFA scores (aHR = 5.45, 95% CI: 3.04–9.79, *p* < 0.001) and late (aHR = 8.99, 95% CI: 4.73–17.06, *p* < 0.001). The floor-effect and early decline groups did not show statistically significant associations with mortality across any of the models.

## Discussion

Sepsis remains a global health challenge with high incidence and mortality, particularly in developing countries where cases and fatalities significantly exceed those in developed regions [4]. Despite therapeutic advances, management consistency and outcomes demand further improvement. The emergency department plays a crucial role in early sepsis care, as clinical data from this setting most accurately reflect the natural disease state, offering critical opportunities for early risk stratification and intervention.

The prognostic value of platelet counts in sepsis remains debated. Although thrombocytopenia is widely recognized as a marker of severity and mortality [7,40,45,46], its independent predictive role is contested [17,47]. Recent studies increasingly focus on dynamic platelet changes—such as platelet trajectory—as potential prognostic indicators [48,49], though evidence remains limited. Further investigation into platelet kinetics is essential to clarify their clinical utility.

In investigating the relationship between platelet parameters and mortality in sepsis, we unexpectedly observed in univariable Cox analysis that Gram-positive and Gram-negative bacterial detection was associated with reduced mortality, whereas other pathogens (including fungi, viruses, and atypical organisms) showed a trend toward increased mortality (HR 1.45, Table 2). These associations persisted after multivariable adjustment in our hierarchical models (Tables 3–6), although they were not the primary focus of this study. Several factors may explain this observation.

First, all patients received early broad-spectrum antibiotics according to Surviving Sepsis Campaign guidelines [16,50], providing effective initial coverage for bacterial infections. Moreover, culture-proven bacterial infections enabled targeted antibiotic adjustment based on susceptibility testing, potentially improving outcomes. Second, non-bacterial pathogens present significant diagnostic challenges. These infections often manifest with nonspecific clinical symptoms and are difficult to detect using conventional culture methods, leading to delayed diagnosis and treatment [51], particularly in immunocompromised patients [52].

Importantly, consistent with the Sepsis-3 consensus definition [1], disease severity—rather than pathogen type—was the strongest predictor of mortality in our cohort. Markers of illness severity (SOFA score, APACHE II score, and lactate level) outperformed pathogen type in prognostic value (Table 2), aligning with large-scale studies demonstrating the predictive utility of SOFA and APACHE II scoresin sepsis outcomes.

In our univariable analysis, platelet nadir and magnitude of platelet decline were also strongly associated with 28-day mortality (both *p* < 0.001, Table 2). To further elucidate the prognostic value of platelet parameters relative to traditional severity scores such as SOFA, APACHE II, and lactate levels, we conducted the following analyses.

Regarding the prognostic value of platelet quantitative parameters—specifically the severity of the platelet nadir and the magnitude of platelet decline—we evaluated both as independent prognostic factors in septic patients, corresponding to our second study objective. These two parameters, while interrelated, capture distinct aspects of platelet dynamics: the platelet nadir reflects the severity of thrombocytopenia achieved during the disease course, whereas the magnitude of platelet decline represents the extent of platelet consumption from baseline. Their combined assessment provides a more comprehensive picture of platelet involvement in sepsis.

First, a clear dose-response relationship was observed between the severity of the platelet nadir and 28-day mortality. After multivariable adjustment, the risk of mortality increased progressively with greater thrombocytopenia severity: compared to patients with normal platelet counts (≥150 × 10⁹/L), the aHR for the moderate (50–99 × 10⁹/L), severe (20–49 × 10⁹/L), and profound (<20 × 10⁹/L) reduction groups were 2.04, 2.98, and 3.46, respectively (all *p* < 0.05, Table 3).

This finding establishes the platelet nadir as an independent risk factor and aligns with existing evidence: Vanderschueren et al [7] identified thrombocytopenia as a stronger predictor of ICU mortality than APACHE II, SAPS II, or MODS scores. Similarly, Claushuis et al [47] reported that platelet counts below 100 × 10⁹/L—particularly in the < 50 and 50–99 × 10⁹/L ranges—were associated with higher illness severity and independently predicted increased 30-day mortality.

Beyond a passive marker, a low platelet nadir may actively contribute to organ dysfunction through microvascular thrombosis, endothelial injury, and immune dysregulation [47]. Severe thrombocytopenia disrupts vascular integrity, weakens immune defense, and promotes microthrombi formation that worsen tissue hypoxia and organ damage [13,53]. This process creates a vicious cycle of platelet consumption and progressive organ injury, ultimately leading to adverse clinical outcomes.

Second, the magnitude of platelet decline was identified as an independent risk factor for 28-day mortality, demonstrating prognostic value beyond the nadir value alone. In our cohort, a clear dose-response relationship was observed: compared to the stable group, patients with severe decline (>50%) exhibited a significantly higher risk of death (aHR = 2.43, *p* < 0.001), and even moderate decline (30–50%) was associated with increased mortality (aHR = 1.79, *p* = 0.044) in the multivariable-adjusted model (Table 4).

This finding aligns with previous studies demonstrating the prognostic value of platelet decline in sepsis, where greater declines are consistently associated with worse outcomes [54]. The magnitude of platelet decline has been proposed as a simple parameter for early risk stratification in critically ill patients [48].

Pathophysiologically, the magnitude of platelet decline reflects the severity of systemic platelet activation and consumption in sepsis [55]. Activated platelets promote leukocyte aggregation and microthrombus formation, while also accelerating their own clearance by the mononuclear phagocyte system in the liver and spleen [56,57]. This process not only shortens platelet lifespan but also perpetuates a vicious cycle of consumption and end-organ injury [58]. Consequently, a greater magnitude of platelet decline serves as an indirect marker of the intensity of immunothrombosis and is associated with increased risks of impaired organ perfusion, tissue hypoxia, and ultimately, adverse outcomes [58].

Turning to our third study objective——the temporal dimension of platelet dynamics——we further investigated whether the timing of the platelet nadir and the timing of the maximum platelet decline were associated with 28-day mortality.

First, regarding the timing of platelet nadir, modest but notable associations were observed. While intermediate and late nadir were significantly associated with increased mortality in standard Cox models (Model 1–3: intermediate aHR = 4.39–4.50, all p < 0.001; late aHR = 5.85–6.09, all p < 0.001), the strength of these associations increased substantially after adjusting for time-updated organ dysfunction scores (Model 4–5: intermediate aHR = 7.16–7.28; late aHR = 9.57–9.58, all p < 0.001), suggesting that delayed platelet recovery captures aspects of persistent disease severity not fully accounted for by baseline adjustments alone. The initial and early nadir groups showed no significant association with mortality across any models (Table 5).

The absence of a significant association between initial or early platelet nadir and mortality warrants further consideration. An initial nadir may reflect pre-existing severe thrombocytopenia present at admission; such patients often present with clinically overt illness that prompts early recognition and aggressive interventions—including early goal-directed therapy and timely antibiotic administration [43]—which may ultimately improve their prognosis. An early nadir (occurring within 2–3 days of admission) likely represents a rapid response to the initial inflammatory storm [40]. Nevertheless, these patients are also readily identifiable in clinical practice and thus more likely to receive timely therapeutic interventions. These findings suggest that the severity of the initial insult does not invariably dictate the final outcome; timely and effective interventions may alter the disease trajectory and interrupt progression from a critical initial state to adverse outcomes [17].

In contrast, an intermediate or late platelet nadir (occurring beyond day 4 of admission) suggests a distinct pathophysiological process. According to the theory of immunological phases in sepsis, the early stage (days 1–3) is characterized by hyperinflammation, whereas the intermediate and late stages (beyond day 4) are marked by immunosuppression, featuring immune cell apoptosis, T-cell exhaustion, and expansion of myeloid-derived suppressor cells [40,44]. During this immunosuppressive phase, sustained inflammatory insults and apoptotic signals may impair megakaryopoiesis in the bone marrow and promote excessive platelet consumption within the microcirculation [59,60]. Concurrently, cytokine imbalances associated with immunosuppression further disrupt the hematopoietic microenvironment [61].

Thus, an intermediate or late platelet nadir not only reflects platelet consumption itself but, more importantly, signifies an integrated failure of the immune-hematopoietic system [62]. Of note, late platelet nadir or maximum platelet decline may also reflect secondary organ failure or a transition to persistent critical illness. This indicates a state of exhausted compensatory mechanisms and cumulative multi-organ injury, characterized by poor response to initial treatment, increased risk of secondary infections, and persistent organ dysfunction, ultimately leading to adverse clinical outcomes [62]. These findings highlight the clinical value of monitoring platelet kinetics: early identification of critically ill patients with sustained platelet decline or delayed recovery may facilitate timely therapeutic adjustments—such as enhanced immune support or optimized anti-infective strategies—thereby improving patient outcomes [16,62].

Second, regarding the timing of maximum platelet decline, both intermediate and late maximum decline were independently associated with increased mortality risk. In standard Cox models (Model 1–3), patients with intermediate decline had an aHR of 2.70–2.98 (all *p* < 0.001), and those with late decline had an aHR of 6.33–6.87 (all *p* < 0.001). After further adjustment for time-updated partial SOFA scores (Model 4–5), these associations were further enhanced (intermediate: aHR = 5.16–5.45; late: aHR = 8.44–8.99, all *p* < 0.001), suggesting that the prognostic impact of delayed maximum decline may be partially independent of concurrent disease severity. The early decline and floor-effect groups showed no significant association with mortality across any of the models.

These findings extend previous observations on the prognostic value of platelet kinetics by demonstrating that not only the magnitude but also the timing of platelet decline independently predicts outcomes in septic patients [47].

The timing of maximum platelet decline extends the findings from nadir timing by incorporating a temporal dimension that better reflects the dynamic nature of sepsis. While nadir timing is restricted to patients who develop thrombocytopenia (nadir <150 × 10⁹/L), maximum decline timing captures both the magnitude and timing of platelet changes across the entire septic cohort—including those whose platelet counts never fall below the thrombocytopenia threshold. This broader applicability enables clinically meaningful comparisons that reflect real-world complexity: for instance, two patients may both experience a 40% decline in platelet count, but if one declines on day 2 and the other on day 7, their prognoses may differ substantially despite similar magnitudes of decline and regardless of whether they ever become thrombocytopenic. Thus, by integrating both the extent and the timing of platelet dynamics, maximum decline timing provides a more comprehensive prognostic tool in the emergency setting, helping to identify high-risk patients—including those who never develop thrombocytopenia—who might otherwise be overlooked.

## Conclusion

This study, through a stepwise design, demonstrates that dynamic platelet changes hold significant prognostic value in sepsis. First, we confirmed that platelet nadir was independently associated with mortality (Table 3), establishing its role in patients with thrombocytopenia. We then extended this finding to the magnitude of platelet decline, which applies to the entire septic cohort but remains a static measure (Table 4). Building on this, we introduced a temporal dimension by examining the timing of platelet nadir, and found that intermediate or late nadir significantly increased mortality risk, suggesting that delayed platelet recovery reflects persistent disease severity and immune-hematopoietic failure (Table 5). Finally, by integrating both magnitude and timing through the timing of maximum platelet decline, this indicator not only captures dynamic changes across all patients but also addresses a question that static measures cannot: whether the same magnitude of decline carries different prognostic implications depending on when it occurs (Table 6). This stepwise investigation reveals that intermediate or late decline indicates dysregulated pathophysiology and depleted compensatory reserves, whereas early decline or early nadir is not associated with mortality—likely benefiting from timely interventions.

From a clinical perspective, the timing of platelet events offers actionable insights: early changes (≤3 days) suggest a reassuring prognosis with continued current management; intermediate changes (4–7 days) signal the need for treatment escalation; and late changes (≥8 days) warrant prognostic counseling and preparation for potential poor outcomes.

In conclusion, these findings underscore the importance of longitudinal platelet monitoring for risk stratification in the emergency setting, as the platelet trajectory integrating both magnitude and timing—rather than any single static value—provides critical prognostic information for identifying patients at risk of poor outcomes, including those who never develop thrombocytopenia.

## Limitations

As a single-center retrospective study, our findings require validation through multi-center research. Although the sample size was sufficient for the primary analysis, we acknowledge that 363 sepsis patients over nearly five years is a relatively modest number, partly attributable to our stringent exclusion criteria, the exclusion of COVID-19 PCR-positive patients, and the competitive healthcare environment with nearby large tertiary hospitals. This modest sample size limited our ability to perform subgroup analyses. Additionally, we did not exclude patients with DNR/DNI status, which may have influenced the results. Consequently, our findings may not be fully generalizable to broader sepsis populations.

Due to the retrospective design, comprehensive longitudinal data on organ dysfunction (e.g., dynamic SOFA, lactate, and ISTH-DIC scores) were not available; we therefore used a time-updated partial SOFA score as a proxy, which may not fully capture the complete trajectory of organ dysfunction. Despite adjusting for a wide range of confounders including lactate levels, residual confounding cannot be fully excluded. Additionally, our study did not systematically collect detailed information on cause of death, limiting our ability to compare whether patients with early versus late platelet decline died from different mechanisms (e.g., refractory septic shock, secondary infections, or withdrawal of care). Future prospective studies incorporating cause-of-death adjudication, along with multi-center studies with larger cohorts and standardized data collection, are warranted to validate our findings and further elucidate the mechanisms underlying the association between platelet kinetics and mortality.

## Supporting information

S1 Data Dataset. **Raw data and derived indicators.xlsx.** This file contains the complete raw patient data and all derived indicators used in the analyses, including demographics, comorbidities, infection characteristics, laboratory parameters, and derived clinical scores (SOFA, APACHE II, ISTH-DIC, and platelet-related parameters).
(XLSX)

S2 Data Dataset. **Serial platelet counts and SOFA scores.xlsx.** This file provides longitudinal platelet measurements and corresponding SOFA scores at baseline, early, intermediate, and late phases, enabling calculation of platelet parameters (nadir, magnitude of decline, timing) and time-updated SOFA scores for Tables 5 and 6.
(XLSX)

S3 Data Dataset. **Excel version of SPSS data for Tables 1–4.xls.** This file contains the analysis-ready dataset derived from S1 Data, formatted for SPSS to replicate the descriptive statistics (Table 1), univariable Cox regression (Table 2), and multivariable Cox regression analyses for platelet nadir (Table 3) and magnitude of platelet decline (Table 4).
(XLS)

S4 Data Dataset. **Excel version of data for** Tables 5**.xlsx.** This file contains the raw dataset prepared for R software to perform time-dependent Cox regression analyses of platelet nadir timing presented in Table 5, including all covariates and time-updated SOFA scores used in Models 1–5.
(XLSX)

S5 Data Dataset. **Excel version of data for** Tables 6**.xlsx.** This file contains the raw dataset prepared for R software to perform time-dependent Cox regression analyses of maximum platelet decline timing presented in Table 6, including all covariates and time-updated SOFA scores used in Models 1–5.
(XLSX)

## References

[1] Singer M, Deutschman CS, Seymour CW, Shankar-Hari M, Annane D, Bauer M, et al. The Third International Consensus Definitions for Sepsis and Septic Shock (Sepsis-3). JAMA. 2016;315(8):801–10. doi: 10.1001/jama.2016.0287 26903338 PMC4968574

[2] Fleischmann-Struzek C, Mellhammar L, Rose N, Cassini A, Rudd KE, Schlattmann P, et al. Incidence and mortality of hospital- and ICU-treated sepsis: results from an updated and expanded systematic review and meta-analysis. Intensive Care Med. 2020;46(8):1552–62. doi: 10.1007/s00134-020-06151-x 32572531 PMC7381468

[3] Fleischmann C, Scherag A, Adhikari NKJ, Hartog CS, Tsaganos T, Schlattmann P, et al. Assessment of Global Incidence and Mortality of Hospital-treated Sepsis. Current Estimates and Limitations. Am J Respir Crit Care Med. 2016;193(3):259–72. doi: 10.1164/rccm.201504-0781OC 26414292

[4] Rudd KE, Johnson SC, Agesa KM, Shackelford KA, Tsoi D, Kievlan DR, et al. Lancet. 2020;395(10219):200–11. doi: 10.1016/S0140-6736(19)32989-7 31954465 PMC6970225

[5] Aird WC. The hematologic system as a marker of organ dysfunction in sepsis. Mayo Clin Proc. 2003;78(7):869–81. doi: 10.4065/78.7.869 12839083

[6] Warkentin TE, Aird WC, Rand JH. Platelet-endothelial interactions: sepsis, HIT, and antiphospholipid syndrome. Hematology Am Soc Hematol Educ Program. 2003;:497–519. doi: 10.1182/asheducation-2003.1.497 14633796

[7] Vanderschueren S, De Weerdt A, Malbrain M, Vankersschaever D, Frans E, Wilmer A, et al. Thrombocytopenia and prognosis in intensive care. Crit Care Med. 2000;28(6):1871–6. doi: 10.1097/00003246-200006000-00031 10890635

[8] Vardon-Bounes F, Ruiz S, Gratacap M-P, Garcia C, Payrastre B, Minville V. Platelets Are Critical Key Players in Sepsis. Int J Mol Sci. 2019;20(14):3494. doi: 10.3390/ijms20143494 31315248 PMC6679237

[9] Ho-Tin-Noé B, Demers M, Wagner DD. How platelets safeguard vascular integrity. J Thromb Haemost. 2011;9 Suppl 1(Suppl 1):56–65. doi: 10.1111/j.1538-7836.2011.04317.x 21781242 PMC3229170

[10] Leone M, Nielsen ND, Russell L. Ten tips on sepsis-induced thrombocytopenia. Intensive Care Med. 2024;50(7):1157–60. doi: 10.1007/s00134-024-07478-5 38739278

[11] Li C, Ture SK, Nieves-Lopez B, Blick-Nitko SK, Maurya P, Livada AC, et al. Thrombocytopenia Independently Leads to Changes in Monocyte Immune Function. Circ Res. 2024;134(8):970–86. doi: 10.1161/CIRCRESAHA.123.323662 38456277 PMC11069346

[12] Cox D. Sepsis - it is all about the platelets. Front Immunol. 2023;14:1210219. doi: 10.3389/fimmu.2023.1210219 37350961 PMC10282552

[13] Clark SR, Ma AC, Tavener SA, McDonald B, Goodarzi Z, Kelly MM, et al. Platelet TLR4 activates neutrophil extracellular traps to ensnare bacteria in septic blood. Nat Med. 2007;13(4):463–9. doi: 10.1038/nm1565 17384648

[14] Anthon CT, Pène F, Perner A, Azoulay E, Puxty K, Van De Louw A, et al. Thrombocytopenia and platelet transfusions in ICU patients: an international inception cohort study (PLOT-ICU). Intensive Care Med. 2023;49(11):1327–38. doi: 10.1007/s00134-023-07225-2 37812225 PMC10622358

[15] Brun-Buisson C, Meshaka P, Pinton P, Vallet B, EPISEPSIS Study Group. EPISEPSIS: a reappraisal of the epidemiology and outcome of severe sepsis in French intensive care units. Intensive Care Med. 2004;30(4):580–8. doi: 10.1007/s00134-003-2121-4 14997295

[16] Evans L, Rhodes A, Alhazzani W, Antonelli M, Coopersmith CM, French C, et al. Surviving Sepsis Campaign: International Guidelines for Management of Sepsis and Septic Shock 2021. Crit Care Med. 2021;49(11):e1063–143. doi: 10.1097/CCM.0000000000005337 34605781

[17] Rhodes A, Evans LE, Alhazzani W, Levy MM, Antonelli M, Ferrer R, et al. Surviving Sepsis Campaign: International Guidelines for Management of Sepsis and Septic Shock: 2016. Intensive Care Med. 2017;43(3):304–77. doi: 10.1007/s00134-017-4683-6 28101605

[18] Ono R, Kitagawa I. SARS-CoV-2 infection-induced immune thrombocytopenia: a systematic review of current reports. Ann Hematol. 2024;103(10):3921–39. doi: 10.1007/s00277-024-05765-1 38652242

[19] Ramlall V, Thangaraj PM, Meydan C, Foox J, Butler D, Kim J, et al. Immune complement and coagulation dysfunction in adverse outcomes of SARS-CoV-2 infection. Nat Med. 2020;26(10):1609–15. doi: 10.1038/s41591-020-1021-2 32747830 PMC7809634

[20] Charlson ME, Pompei P, Ales KL, MacKenzie CR. A new method of classifying prognostic comorbidity in longitudinal studies: development and validation. J Chronic Dis. 1987;40(5):373–83. doi: 10.1016/0021-9681(87)90171-8 3558716

[21] Suidan RS, Leitao MM Jr, Zivanovic O, Gardner GJ, Long Roche KC, Sonoda Y, et al. Predictive value of the Age-Adjusted Charlson Comorbidity Index on perioperative complications and survival in patients undergoing primary debulking surgery for advanced epithelial ovarian cancer. Gynecol Oncol. 2015;138(2):246–51. doi: 10.1016/j.ygyno.2015.05.034 26037900 PMC4972341

[22] Pan Y, Liu Z-P, Dai H-S, Chen W-Y, Luo Y, Wang Y-Z, et al. Development of a model based on the age-adjusted Charlson comorbidity index to predict survival for resected perihilar cholangiocarcinoma. World J Gastrointest Oncol. 2023;15(6):1036–50. doi: 10.4251/wjgo.v15.i6.1036 37389112 PMC10302988

[23] Fierro-Angulo OM, González-Regueiro JA, Pereira-García A, Ruiz-Margáin A, Solis-Huerta F, Macías-Rodríguez RU. Hematological abnormalities in liver cirrhosis. World J Hepatol. 2024;16(9):1229–44. doi: 10.4254/wjh.v16.i9.1229 39351511 PMC11438588

[24] Zanetto A, Campello E, Senzolo M, Simioni P. The evolving knowledge on primary hemostasis in patients with cirrhosis: A comprehensive review. Hepatology. 2024;79(2):460–81. doi: 10.1097/HEP.0000000000000349 36825598

[25] Kaw D, Malhotra D. Platelet dysfunction and end-stage renal disease. Semin Dial. 2006;19(4):317–22. doi: 10.1111/j.1525-139X.2006.00179.x 16893410

[26] Shah R, Haddad N, Vachharajani TJ, Asif A, Agarwal A. Thrombocytopenia in ESRD patients: epidemiology, mechanisms and interventional nephrology perspective. Semin Dial. 2014;27(6):618–25. doi: 10.1111/sdi.12199 24612107

[27] Vincent JL, Moreno R, Takala J, Willatts S, De Mendonça A, Bruining H, et al. The SOFA (Sepsis-related Organ Failure Assessment) score to describe organ dysfunction/failure. On behalf of the Working Group on Sepsis-Related Problems of the European Society of Intensive Care Medicine. Intensive Care Med. 1996;22(7):707–10. doi: 10.1007/BF01709751 8844239

[28] Knaus WA, Draper EA, Wagner DP, Zimmerman JE. APACHE II: a severity of disease classification system. Crit Care Med. 1985;13(10):818–29. 3928249

[29] Taylor FB Jr, Toh CH, Hoots WK, Wada H, Levi M, Scientific Subcommittee on Disseminated Intravascular Coagulation (DIC) of the International Society on Thrombosis and Haemostasis (ISTH). Towards definition, clinical and laboratory criteria, and a scoring system for disseminated intravascular coagulation. Thromb Haemost. 2001;86(5):1327–30. doi: 10.1055/s-0037-1616068 11816725

[30] George JN, Raskob GE, Shah SR, Rizvi MA, Hamilton SA, Osborne S, et al. Drug-induced thrombocytopenia: a systematic review of published case reports. Ann Intern Med. 1998;129(11):886–90. doi: 10.7326/0003-4819-129-11_part_1-199812010-00009 9867731

[31] Zhu Y, Huang L, Zhang J, Liang L, Jin P. Incidence and risk factors of Vancomycin-induced thrombocytopenia: a six-year real-world study. BMC Infect Dis. 2025;25(1):7. doi: 10.1186/s12879-024-10393-1 39748286 PMC11697819

[32] Howard CE, Adams LA, Admire JL, Chu MA, Alred GL. Vancomycin-induced thrombocytopenia: a challenge and rechallenge. Ann Pharmacother. 1997;31(3):315–8. doi: 10.1177/106002809703100308 9066938

[33] Lin S-Y, Huang J-C, Shen M-C, Chuang S-H, Lee M-H, Chen H-C. Piperacillin-induced thrombocytopenia reversed by high-flux hemodialysis in an uremic patient. Hemodial Int. 2012;16 Suppl 1:S50-3. doi: 10.1111/j.1542-4758.2012.00745.x 23036037

[34] Alzahrani M, Alrumaih I, Alhamad F, Abdel Warith A. Rapid onset severe thrombocytopenia following reexposure to piperacillin-tazobactam: report of two cases and review of the literature. Platelets. 2018;29(6):628–31. doi: 10.1080/09537104.2018.1468025 29863943

[35] Huang R, Cai G-Q, Zhang J-H, Liu F-X, Ma J-Q, Liu H, et al. Meropenem-induced immune thrombocytopenia and the diagnostic process of laboratory testing. Transfusion. 2017;57(11):2715–9. doi: 10.1111/trf.14267 28782250

[36] Qiao W, Chang C, Wang Q, Cao X, Zhang X. Imipenem cilastatin sodium-associated thrombocytopenia in an older patient: A case report and literature review. Int J Clin Pharmacol Ther. 2022;60(8):358–63. doi: 10.5414/CP204215 35652550

[37] Domingo-Chiva E, Díaz-Rangel M, Monsalve-Naharro JÁ, Cuesta-Montero P, Catalá-Ripoll JV, García-Martínez EM. Ceftazidime-induced thrombocytopenia. Rev Esp Anestesiol Reanim. 2017;64(10):590–3. doi: 10.1016/j.redar.2017.03.016 28559046

[38] Aryal B, Simmons A, Ekkurthi G, Panthi B, Pena-Vizcarra OR. Ceftriaxone-Induced Thrombocytopenia: A Case Report on a Rare Medical Condition. Cureus. 2025;17(5):e83811. doi: 10.7759/cureus.83811 40497213 PMC12151523

[39] Wang M-G, Wang D, He J-Q. Reversible recurrent profound thrombocytopenia due to linezolid in a patient with multi-drug resistant tuberculosis: A case report. Medicine (Baltimore). 2018;97(34):e11997. doi: 10.1097/MD.0000000000011997 30142839 PMC6113018

[40] Akca S, Haji-Michael P, de Mendonça A, Suter P, Levi M, Vincent JL. Time course of platelet counts in critically ill patients. Crit Care Med. 2002;30(4):753–6. doi: 10.1097/00003246-200204000-00005 11940740

[41] Greinacher A, Selleng K. Thrombocytopenia in the intensive care unit patient. Hematology Am Soc Hematol Educ Program. 2010;2010:135–43. doi: 10.1182/asheducation-2010.1.135 21239783

[42] Cuker A, Gimotty PA, Crowther MA, Warkentin TE. Predictive value of the 4Ts scoring system for heparin-induced thrombocytopenia: a systematic review and meta-analysis. Blood. 2012;120(20):4160–7. doi: 10.1182/blood-2012-07-443051 22990018 PMC3501714

[43] Rivers E, Nguyen B, Havstad S, Ressler J, Muzzin A, Knoblich B, et al. Early goal-directed therapy in the treatment of severe sepsis and septic shock. N Engl J Med. 2001;345(19):1368–77. doi: 10.1056/NEJMoa010307 11794169

[44] Hotchkiss RS, Monneret G, Payen D. Sepsis-induced immunosuppression: from cellular dysfunctions to immunotherapy. Nat Rev Immunol. 2013;13(12):862–74. doi: 10.1038/nri3552 24232462 PMC4077177

[45] Mavrommatis AC, Theodoridis T, Orfanidou A, Roussos C, Christopoulou-Kokkinou V, Zakynthinos S. Coagulation system and platelets are fully activated in uncomplicated sepsis. Crit Care Med. 2000;28(2):451–7. doi: 10.1097/00003246-200002000-00027 10708182

[46] Pène F, Russell L, Aubron C. Thrombocytopenia in the intensive care unit: diagnosis and management. Ann Intensive Care. 2025;15(1):25. doi: 10.1186/s13613-025-01447-x 39985745 PMC11846794

[47] Claushuis TAM, van Vught LA, Scicluna BP, Wiewel MA, Klein Klouwenberg PMC, Hoogendijk AJ, et al. Thrombocytopenia is associated with a dysregulated host response in critically ill sepsis patients. Blood. 2016;127(24):3062–72. doi: 10.1182/blood-2015-11-680744 26956172

[48] Moreau D, Timsit J-F, Vesin A, Garrouste-Orgeas M, de Lassence A, Zahar J-R, et al. Platelet count decline: an early prognostic marker in critically ill patients with prolonged ICU stays. Chest. 2007;131(6):1735–41. doi: 10.1378/chest.06-2233 17475637

[49] Strauss R, Wehler M, Mehler K, Kreutzer D, Koebnick C, Hahn EG. Thrombocytopenia in patients in the medical intensive care unit: bleeding prevalence, transfusion requirements, and outcome. Crit Care Med. 2002;30(8):1765–71. doi: 10.1097/00003246-200208000-00015 12163790

[50] Dellinger RP, Levy MM, Rhodes A, Annane D, Gerlach H, Opal SM, et al. Surviving sepsis campaign: international guidelines for management of severe sepsis and septic shock: 2012. Crit Care Med. 2013;41(2):580–637. doi: 10.1097/CCM.0b013e31827e83af 23353941

[51] Perfect JR. Fungal diagnosis: how do we do it and can we do better?. Curr Med Res Opin. 2013;29 Suppl 4:3–11. doi: 10.1185/03007995.2012.761134 23621588

[52] Fang W, Wu J, Cheng M, Zhu X, Du M, Chen C, et al. Diagnosis of invasive fungal infections: challenges and recent developments. J Biomed Sci. 2023;30(1):42. doi: 10.1186/s12929-023-00926-2 37337179 PMC10278348

[53] Kroll MH, Afshar-Kharghan V. Platelets in pulmonary vascular physiology and pathology. Pulm Circ. 2012;2(3):291–308. doi: 10.4103/2045-8932.101398 23130099 PMC3487299

[54] Schupp T, Weidner K, Rusnak J, Jawhar S, Forner J, Dulatahu F, et al. Diagnostic and prognostic role of platelets in patients with sepsis and septic shock. Platelets. 2023;34(1):2131753. doi: 10.1080/09537104.2022.2131753 36484263

[55] Greco E, Lupia E, Bosco O, Vizio B, Montrucchio G. Platelets and Multi-Organ Failure in Sepsis. Int J Mol Sci. 2017;18(10):2200. doi: 10.3390/ijms18102200 29053592 PMC5666881

[56] McGregor L, Martin J, McGregor JL. Platelet-leukocyte aggregates and derived microparticles in inflammation, vascular remodelling and thrombosis. Front Biosci. 2006;11:830–7. doi: 10.2741/1840 16146774

[57] Grozovsky R, Hoffmeister KM, Falet H. Novel clearance mechanisms of platelets. Curr Opin Hematol. 2010;17(6):585–9. doi: 10.1097/MOH.0b013e32833e7561 20729731 PMC4303238

[58] Assinger A, Schrottmaier WC, Salzmann M, Rayes J. Platelets in Sepsis: An Update on Experimental Models and Clinical Data. Front Immunol. 2019;10:1687. doi: 10.3389/fimmu.2019.01687 31379873 PMC6650595

[59] François B, Trimoreau F, Vignon P, Fixe P, Praloran V, Gastinne H. Thrombocytopenia in the sepsis syndrome: role of hemophagocytosis and macrophage colony-stimulating factor. Am J Med. 1997;103(2):114–20. doi: 10.1016/s0002-9343(97)00136-8 9274894

[60] Dewitte A, Lepreux S, Villeneuve J, Rigothier C, Combe C, Ouattara A, et al. Blood platelets and sepsis pathophysiology: A new therapeutic prospect in critically [corrected] ill patients?. Ann Intensive Care. 2017;7(1):115. doi: 10.1186/s13613-017-0337-7 29192366 PMC5709271

[61] de Melo TC, Ariga SKK, de Lima TM, Levy D, Bydlowski SP, Soriano FG. Impact of sepsis on bone marrow mesenchymal stem cells and its implications for hematopoiesis and immunosuppression. Inflamm Res. 2025;74(1):115. doi: 10.1007/s00011-025-02083-8 40879757

[62] Girardis M, David S, Ferrer R, Helms J, Juffermans NP, Martin-Loeches I, et al. Understanding, assessing and treating immune, endothelial and haemostasis dysfunctions in bacterial sepsis. Intensive Care Med. 2024;50(10):1580–92. doi: 10.1007/s00134-024-07586-2 39222142

